# Triboelectric effect-modulated varifocal liquid lens

**DOI:** 10.1038/s41378-020-0174-y

**Published:** 2020-08-10

**Authors:** Chunlong Fang, Yuanzhi Cao, Dongdong Jiang, Jiarui Tian, Chi Zhang

**Affiliations:** 1 CAS Center for Excellence in Nanoscience, Beijing Key Laboratory of Micro-nano Energy and Sensor, Beijing Institute of Nanoenergy and Nanosystems, Chinese Academy of Sciences, 100083 Beijing, P.R. China; 2 School of Nanoscience and Technology, University of Chinese Academy of Sciences, 100049 Beijing, P.R. China; 3 Center on Nanoenergy Research, School of Physical Science and Technology, Guangxi University, 530004 Nanning, P.R. China

**Keywords:** Other nanotechnology, Engineering

## Abstract

Electrically modulated varifocal liquid lenses, which are usually modulated by an external high voltage power source, have attracted much attention due to their bright application prospects in artificial optical systems. Here, a triboelectric nanogenerator (TENG)-based varifocal liquid lens (TVLL) has been demonstrated, in which the focal length can be directly modulated by external mechanical sliding. A dielectrophoretic force is generated by the TENG through the transfer of triboelectric charges in the asymmetric electrodes, which is used to continuously change the shape of the air–liquid interface between concave and convex without any complicated boost converter. Moreover, a triboelectric magnifying glass based on the accurate modulation effect of the TVLL on a light beam has been demonstrated. In this work, the TENG is used as a medium to modulate and accurately control the focal length of the liquid lens by an external mechanical stimulus, which may have great applications in micro-optical-electro-mechanical systems (MOEMS), human–machine interaction, artificial vision systems, etc.

## Micro-optical systems: Novel liquid lens controlled by contact electrification

The triboelectric effect is a type of contact electrification, and a liquid lens with variable focus has been developed using a triboelectric nanogenerator (TENG) where the focal length can be directly controlled by external mechanical sliding. With most artificial optical systems, adjusting the focal length is achieved by varying the spacing between the lenses, which results in complex mechanical designs and demands considerable space. However, a team headed by Chi Zhang at the Chinese Academy of Sciences, Beijing succeeded in producing a TENG-based varifocal liquid lens (TVLL), in which the TENG is used to accurately control the focal length of the lens by means of an external mechanical stimulus. The authors believe that their TVLL has excellent potential for application in such areas as micro-optical electromechanical systems, human-machine interactions, and artificial vision systems.

## Introduction

For most artificial optical systems, focal length tuning is accomplished by varying the spacing between lenses; thus, a complicated mechanical design and a large space are indispensable. This is not in accord with the development trend of simple structures and small sizes for next-generation portable optical devices. In recent years, the varifocal liquid lens has attracted great interest due to its remarkable merits, such as a wide adjustment range, a simple structure, and a small size^1–5^. According to the modulation mode, varifocal liquid lenses can be classified into two categories. The first mode corresponds to mechanically modulated liquid lenses whose focal lengths are modulated by changing the volume of the liquid in the lens chamber by using a fluid pumping system^6–8^. The operation mechanism is simple, but it is sensitive to vibrations in the surroundings and inconvenient for portable devices. The other mode corresponds to the electrically modulated liquid lenses whose focal lengths are modulated by an external high voltage^9–12^. The advantages of this mode are its wide adjustment range and lack of a complex mechanical moving part. However, the high voltage required for modulation presents new challenges in terms of power consumption and safety.

In 2012, the triboelectric nanogenerator (TENG) was invented by Wang’s group, which was derived from the second term of the Maxwell displacement current^13–16^. A TENG can effectively convert tiny mechanical energy into electricity with high voltage and low current. Recently, TENGs have successfully served as novel and safe high voltage supplies for various functional devices/systems, such as tunable MEMS mirrors^17^, triboelectric micromotors^18^, liquid robots^19^, microplasmas^20^, electrospinning systems^21^, microfluidic devices^22,23^, mass spectrometers^24^, and air cleaners^25,26^. Therefore, a TENG as a controllable high-voltage power supply would provide an ideal solution to modulate a varifocal liquid lens by an external mechanical stimulus, without any external power supply or complicated boost converter.

In this work, we developed a triboelectric effect-modulated varifocal liquid lens (TVLL) in which the focal length can be directly modulated by external mechanical sliding. A dielectrophoretic force is generated by the TENG through the transfer of triboelectric charges in the asymmetric electrodes, which is used to continuously change the shape of the air–liquid interface between concave and convex without any complicated boost converter. Moreover, a triboelectric magnifying glass was demonstrated based on the accurate modulation effect of the TVLL on a light beam. In this work, the TENG is used as a medium to modulate and accurately control the focal length of the liquid lens by an external mechanical stimulus, which may have great application potential in micro-optical-electro-mechanical systems (MOEMS), human–machine interaction, artificial vision systems, etc.

## Results and discussions

### Overview of the TVLL

**Fig. 1:**
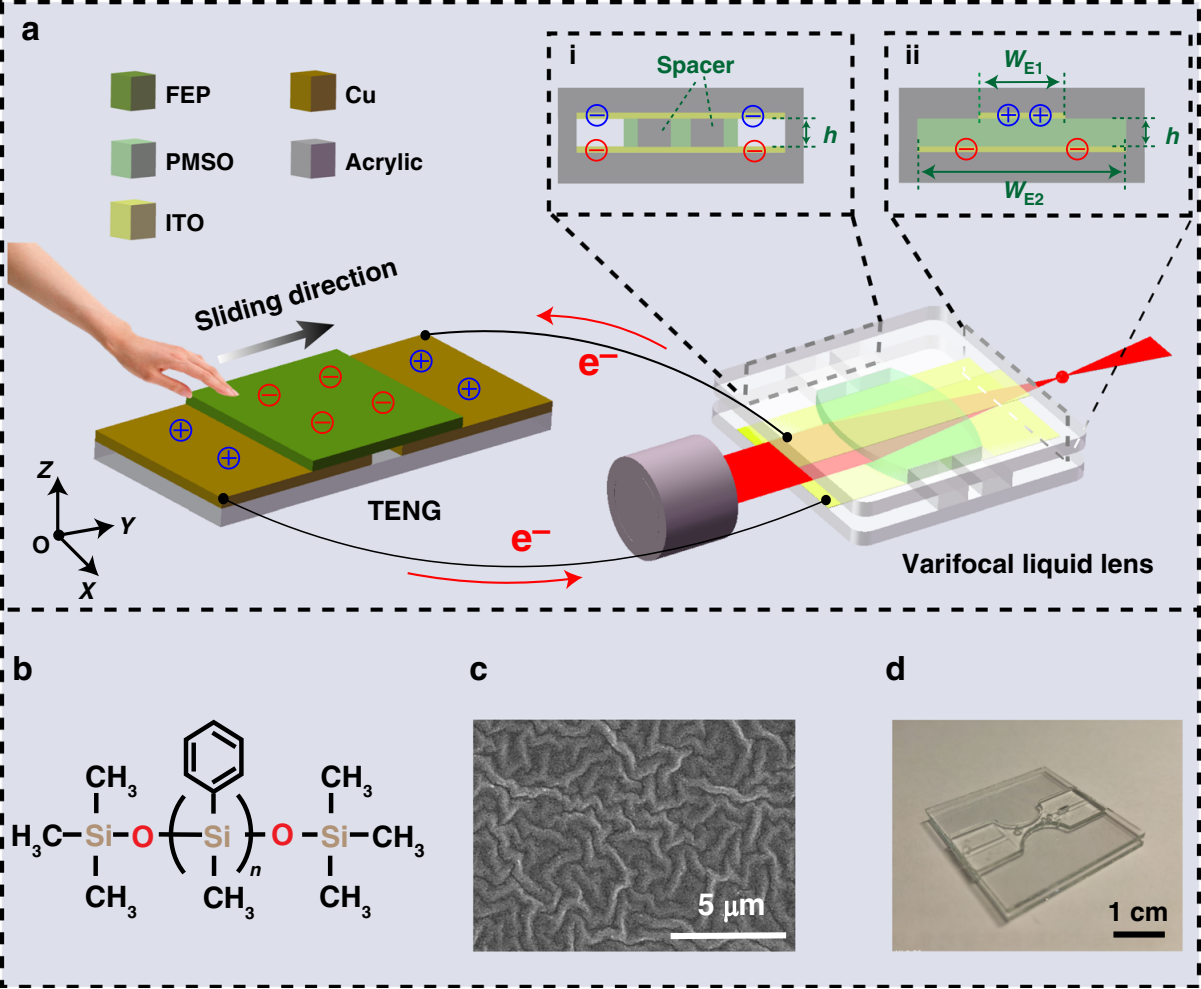
Overview of the triboelectric effect-modulated varifocal liquid lens (TVLL). **a** Schematic diagram of the TVLL. **b** Molecular structure of the PMSO applied in the TVLL. **c** SEM image of the FEP surface with etched micro/nanostructures. **d** Photograph of the fabricated TVLL

The basic structure of the TVLL consists of a freestanding-mode TENG and a varifocal liquid lens with a sandwich structure, in which the two electrodes of the TENG are directly connected to the upper and lower electrodes of the liquid lens (Fig. 1a). The proposed liquid lens has a sandwich structure, and the function of the spacer is to form an optical liquid chamber with upper and lower PMMA substrates. The length, width, and height of the liquid lens chamber are 10, 10, and 1 mm, respectively. As the sliding triboelectric layer of the TENG is moved by external mechanical stimuli, charges are transferred from the TENG to the asymmetric electrodes of the liquid lens, and the shape of the liquid lens is continuously modulated from biconcave to planar and eventually to biconvex by the dielectrophoretic forces generated by the heterogeneous electric field. For the varifocal liquid lens, the asymmetric electrodes are the key components that determine whether the focal length can be modulated by the TENG (Fig. 1a.i and ii shows cross-sections of the liquid lens). In our study, the asymmetric electrodes consist of two parallel plate electrodes with different widths and equal lengths. By applying triboelectric charges of opposite polarity on the two parallel plate electrodes, an inhomogeneous electric field is formed that gradually decays from the center of the two parallel plate electrodes to the edges. It is worth noting that the inhomogeneous electric field is axisymmetric along the length of the electrodes. This means that the dielectrophoretic forces acting on the two air–liquid interfaces of the liquid lens are completely equal, achieving a dual-sided liquid lens with a wide adjustment range. Moreover, this structural design also has the advantages of easy fabrication and low cost.

For the TENG, two dissimilar materials, copper and fluorinated ethylene propylene (FEP), are selected as the frictional layers for triboelectrification. Specifically, the surface of the FEP film was etched with macro/nanostructures (Fig. 1c) by inductively coupled plasma (ICP), which could increase the effective surface area and thus effectively enhance the output performance of the TENG.

Polyphenylmethylsiloxane (PMSO) was selected as the optical medium due to its unique attributes, such as a high transmittance (over 90% to visible light), a slow evaporation rate, a low viscous resistance and excellent electrical insulation performance. Its molecular structure is shown in Fig. 1b. A digital photograph of the fabricated varifocal liquid lens is shown in Fig. 1d.

### Working principle of the TVLL

**Fig. 2:**
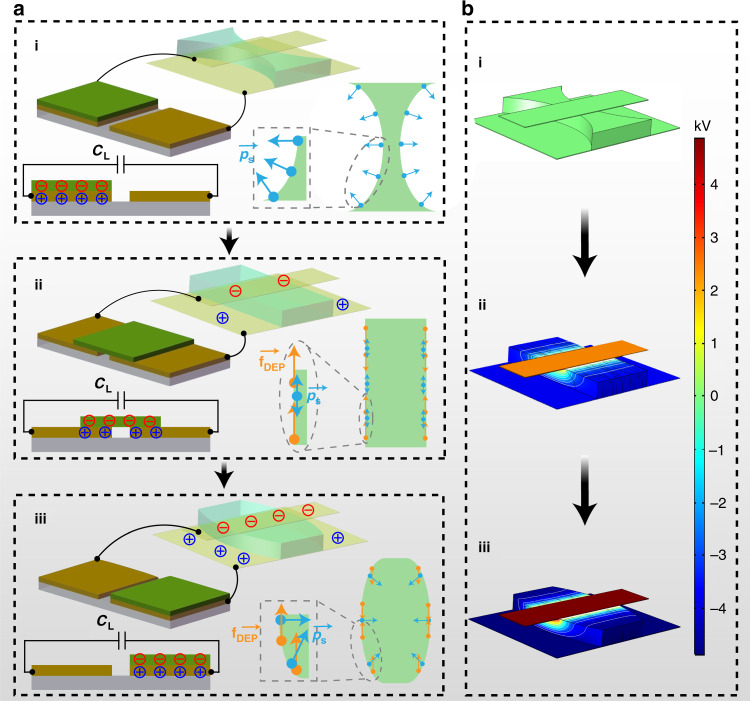
Working principle of the TVLL for continuous variation of the focal length. **a** Focal length variation for three typical states: i biconvex, ii planar, and iii biconcave. The inserts show the force analysis of the corresponding state (the blue arrows represents the pressure differences, *p*_s_; the orange arrows represents the dielectrophoretic force, *f*_DEP_). **b** Corresponding numerical calculation of the electric potential distribution on the TVLL

The detailed working principle of continuous variation of the focal length can be divided into two processes, as shown in Fig. 2a. Initially, the sliding triboelectric layer (FEP) fully contacts the left electrode of the TENG (Fig. 2a.i). There is an equal amount of opposite charges on their contact surfaces, according to the triboelectric series that copper more easily loses electrons than FEP. In this state, charges have not been transferred to the asymmetric electrodes. The shape of the liquid lens is biconcave (Fig. 2a.i), which is determined by the surface tension. According to the Laplace law, the pressure difference at the air–liquid interface can be expressed as^27^

1 $$\Delta P_{\mathrm {s}} = \gamma k$$

where Δ*P*_s_ is the pressure difference, *γ* is the surface tension between air and the liquid, and *κ* is the curvature of the air–liquid interface.

Then, when the FEP layer is moved by external mechanical sliding (Fig. 2a.ii), electrons from one of the asymmetric electrodes are injected into the left electrode of the TENG, while electrons from the right electrode can flow into the other asymmetric electrode. The opposite charges on the asymmetric electrodes form an inhomogeneous electric field. The air–liquid interface in the inhomogeneous electric field is subjected to dielectrophoretic forces that can manipulate the liquid toward where the electric field is strong and away from where the electric field is weak.

The dielectrophoretic forces (*F*_DEP_) can be expressed as^12^

2 $$F_{\mathrm {{DEP}}} = \frac{{\varepsilon _0(\varepsilon _{\mathrm {L}} - 1)w}}{{2h}}V^2$$

where *ε*_0_ is the permittivity of vacuum, equal to 8.85 × 10^−12^ F/m, *ε*_L_ (*ε*_L_ = 2.5) is the relative permittivity of PMSO, *V* is the applied voltage, and *w* and *h* are the width and gap of the electrodes, respectively. Apparently, for a given fabricated device, the magnitude of the variation is determined by the applied voltage, as indicated in Fig. 2a.i–iii. Therefore, the sliding distance of the triboelectric layer (FEP) of the TENG can form an interactive relationship with the focal length of the liquid lens.

The relevant numerical calculation of the electric potential distribution on the asymmetric electrodes of the TVLL by the finite-element method (FEM) in COMSOL 5.4 software was simplified as illustrated in Fig. 2b. The simulation results indicate that the intensity of the electric field on the asymmetric electrodes of the TVLL is gradually enhanced as the FEP layer moves from the left to the right electrode of the TENG, well consistent with the actual working process.

### Characteristics of the TENG

**Fig. 3:**
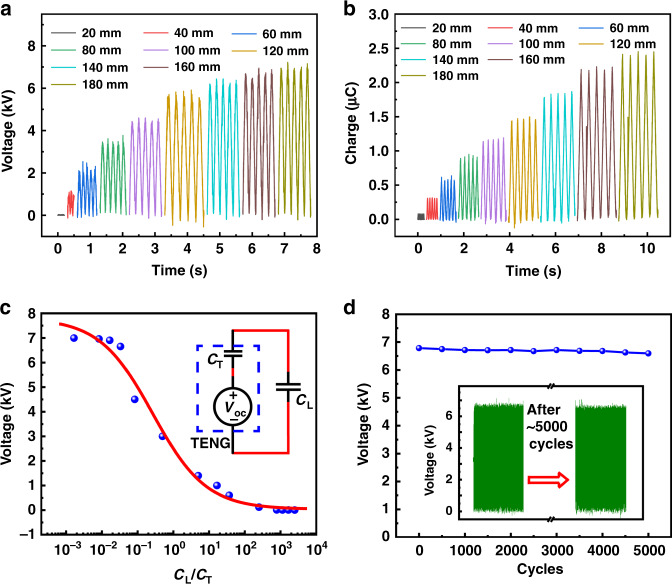
Characteristics of the TENG applied for the TVLL. **a**, **b** Open-circuit voltage and transferred charges of the TENG for different sliding distances. **c** Dependence of the output voltage of the TENG on the loading capacitor. The inset shows the equivalent electric circuit diagram. **d** Durability of the TENG over various cycles

The output performance of the freestanding-mode TENG was characterized, as shown in Fig. 3. Figure 3a, b indicates that as the sliding distance increases from 0 to 180 mm, the open-circuit voltage and short-circuit transferred charges almost linearly increase from 0 to 7 kV and from 0 to 2.4 μC, respectively. At the same time, we obtained the *V* output vs. sliding distance in the TENG by FEM simulation, and the result (Fig. S3) is in good agreement with the experimental measurement (Fig. 3a) and previous work^28^, confirming the accuracy of the measurement. The proposed varifocal liquid lens is a typical capacitive device. Thus, the output characteristics of the TENG when connected to a commercial capacitor were investigated, as shown in Fig. 3c. According to previous work^29^, the freestanding-mode TENG is equivalent to an ideal high voltage source (*V*_OC_) and a constant capacitor (*C*_T_) in series, and the equivalent circuit diagram is shown in Fig. 3c (inset). The results indicate that the voltage applied on *C*_L_ varies as the ratio of *C*_L_ to *C*_T_ varies, especially in the range of 10^−1^–10^1^. Finally, the stability of the output of the TENG (maximum open-circuit voltage) was also investigated (Fig. 3d). The measurement results (inset in Fig. 3d) indicate that there is only a slight decay of the maximum open-circuit voltage during continuous operation for 5000 cycles. Therefore, the TENG could serve as a reliable high voltage power supply for modulation of a varifocal liquid lens.

### Characteristics of the TVLL

**Fig. 4:**
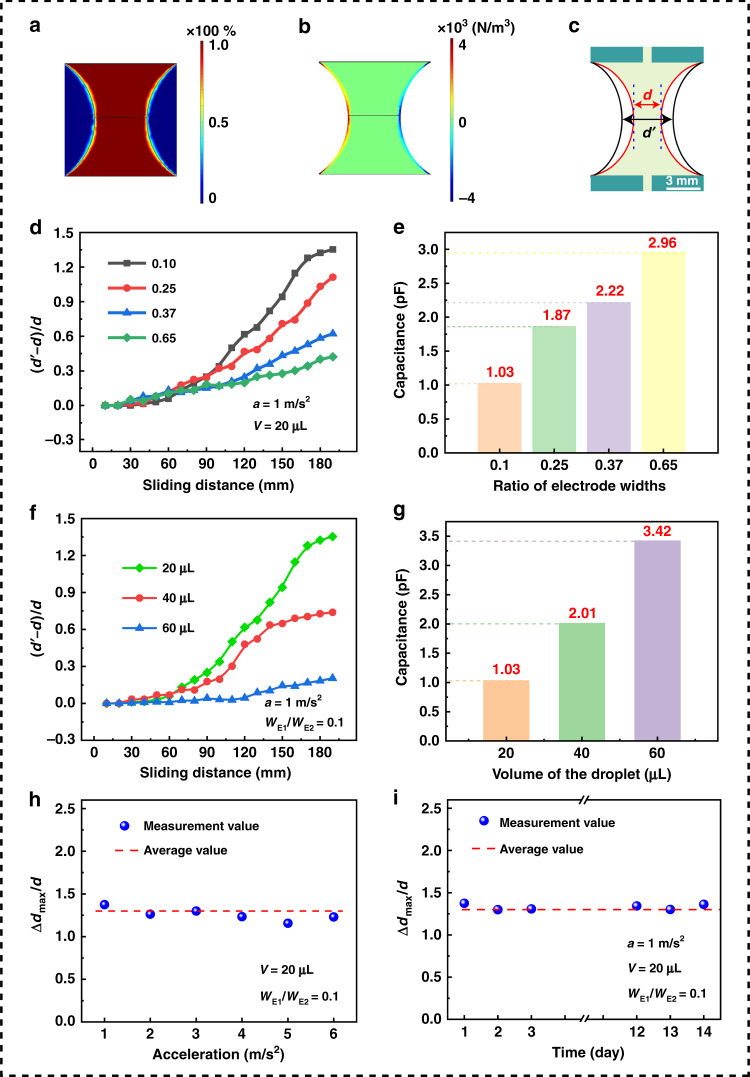
Characteristics of the TVLL with varying focal length. **a**, **b** Distribution of the composition and dielectrophoretic force of PMSO. **c** Defined parameters of the TVLL for measuring the variation. **d** Variation characteristics for different electrode width ratios. **e** Capacitance corresponding to **d**. **f** Variation characteristics for different droplet volumes. **g** Capacitance corresponding to **f**. **h** Variation characteristics for different sliding velocities (accelerations). **i** Durability of the TVLL over 2 weeks

As discussed in the working principle section, a dielectrophoretic force is exerted on the air–liquid interface. Thus, the air–liquid interface was first analyzed by using the FEM in COMSOL 5.4 software. As shown in Fig. 4a, the air–liquid interface is not a strict interface but a thin two-phase mixing zone. Thus, the dielectrophoretic force exists at a certain depth of the liquid droplet, as shown in Fig. 4b.

To visually and conveniently describe the change in the liquid shape, the distance (*d*) between the two air–liquid interfaces was chosen as the parameter for analysis, as shown in Fig. 4c. First, the influence of the width of the electrodes on the deformation of the air–liquid interface was investigated. As the experimental results in Fig. 4d show, for a certain width of the electrodes, as the sliding distance increases, *d* increases. This occurs because with increasing sliding distance of the TENG, the voltage applied on the liquid lens increases, and according to Eq. (2), the dielectrophoretic force is increased. In other words, a larger sliding distance of the TENG means a larger dielectrophoretic force exerted on the air–liquid interface and a larger deformation. For a certain sliding distance of the TENG, when one electrode width is fixed (*W*_E2_ = 10 mm), as the other electrode width (*W*_E1_) increases, *d* decreases. This is due to the increase in *W*_E1_ resulting in an increase in the equivalent capacitance of the liquid lens (*C*_L_), as the experimental measurement in Fig. 4e shows. According to the experimental results shown in Fig. 3c in the previous section, as *C*_L_ increases (*C*_T_ is a constant, ~6.63 pF), the voltage applied on the liquid lens decreases, which means that a smaller dielectrophoretic force is exerted on the air–liquid interface, and a smaller deformation is obtained. Next, the influence of the volume of the droplet on the deformation of the air–liquid interface was investigated. As the experimental measurement illustrated in Fig. 4f shows, for a certain sliding distance of the TENG, as the volume increases, *d* decreases. This occurs because with increasing volume, the equivalent capacitance of the liquid lens (*C*_L_) increases, as the experimental measurement in Fig. 4g shows. In addition, the voltage applied on the liquid lens decreases, which means that a smaller dielectrophoretic force is exerted on the air–liquid interface, resulting in a smaller deformation. Then, the influence of the sliding speed (acceleration, *a*) on the deformation of the air–liquid interface was investigated. As the experimental measurement illustrated in Fig. 4h shows, for a certain sliding distance of the TENG, the deformation (*d*) is almost constant with increasing sliding speed (*a*). This occurs because the output voltage of the freestanding-mode TENG is determined by the triboelectric charge quantity and sliding distance (contact area) but is independent of the sliding velocity^17,28^. This means that the dielectrophoretic force exerted on the air–liquid interface does not change, and thus, the deformation (*d*) is constant. Finally, a stability test of the deformation of the air–liquid interface was performed with a sliding distance of 180 mm, an electrode width ratio of 0.1, a droplet volume of 20 μL and a sliding speed of 1 m/s^2^, as presented in Fig. 4i, which indicates that the fabricated TVLL has good long-term stability for further application.

The equivalent circuit diagram shown in Fig. 3c (inset) can be regarded as a TENG powering a capacitive device. According to a previous work^30,31^, the transferred triboelectric charges can be maintained in the load capacitive device, which means that the state of the liquid lens can be sustained. In our experiments, when the liquid lens is modulated by the TENG, it can be in a stable state. However, because of the imperfect device fabrication and because the selected materials are not completely insulated, there is gradual leakage of the transferred triboelectric charges. This issue could be solved by optimizing the fabrication technology and enhancing the insulating properties of the materials.

### Modulation effect on a light beam

**Fig. 5:**
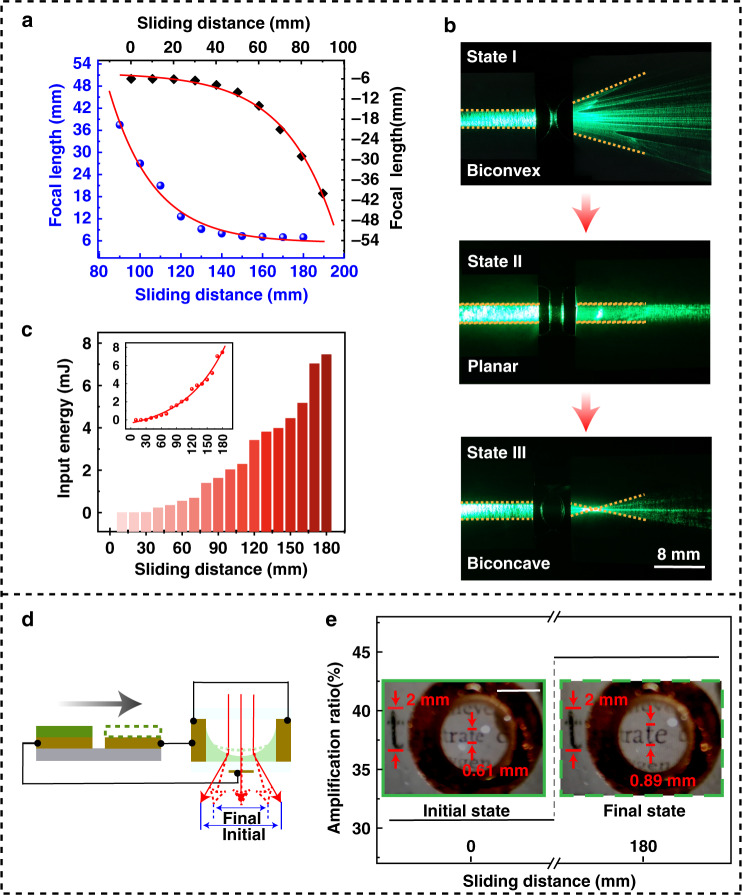
Modulation effect on a light beam. **a** Relationship between the sliding distance of the TENG and focal length. **b** Photograph of a laser beam modulated by the TVLL. **c** Energy consumed in the modulation process. **d** Proposed novelty magnifying glass based on the TVLL. **e** Magnification corresponding to different states (scale bar, 2 mm)

For the liquid lens, realizing focal length variation over a wide range without any complicated mechanical assistance system is its greatest advantage. Here, the relationship between the focal length and sliding distance of the TENG was investigated. As the experimental measurement illustrated in Fig. 5a shows, with the sliding distance increasing from zero to the maximum, the focal length of the liquid lens is continuously modulated from −6 mm to infinite to 7 mm. The modulation process consists of two parts: as the sliding distance increases from 0 to 90 mm, the shape of the liquid lens is modulated from biconvex to planar, and the corresponding focal length of the liquid lens is changed from −6 mm to infinite, as shown in the upper right part of Fig. 5a; as the sliding distance increases from 90 to 180 mm, the shape of the liquid lens is modulated from planar to biconcave, and the corresponding focal length is changed from infinite to 7 mm, as shown in the lower left part of Fig. 5a. The experimental results were fitted by the Origin embedded function *ExpDec2*, which well described the relationship between the sliding distance of the TENG and the focal length of the liquid lens with an *Adj. R-Square* of over 0.99. It is worth noting that there is a one-to-one correspondence between the sliding distance and the focal length, which means that the focal length could be accurately controlled by changing the sliding distance of the TENG. In our experiment, both the human hand-controlled and linear motor-controlled TENG demonstrated a perfect modulation effect on the liquid lens. Considering the accuracy of the results, when the TENG was controlled by the linear motor, the velocity was 0.1 m/s. In other words, an interesting human–machine (light) interaction relationship was obtained, which has broad application prospects in MOEMS, portable optical devices/systems, etc. Figure 5b exhibits the modulation effect of the TVLL on a laser beam. Meanwhile, the energy consumption during the modulation process was also evaluated. The experimental measurement is shown in Fig. 5c; due to the high voltage and low current output characteristics, the energy consumption is as low as the millijoule level. This means that the proposed TVLL does not pose a safety problem compared to the traditional electrically modulated varifocal liquid lens. On this basis, a triboelectric magnifying glass was demonstrated. The schematic diagram is shown in Fig. 5d, in which the curvature of the air–liquid interface could be modulated by the TENG, which eventually presented as a change in the magnification ratio. As the experimental results in Fig. 5e and Video S2 show, as the sliding distance of the TENG increases from zero (initial state) to 180 mm (final state), the height of the letter “t” in the image increases from 0.61 to 0.89 mm. Compared to the actual height of 2 mm, the magnification ratio is modulated from 30.7% to 44.6%.

## Conclusions

In summary, we reported a TVLL realized by coupling a liquid lens and a TENG, in which the focal length could be directly modulated by external mechanical sliding. A dielectrophoretic force is generated by the TENG through the transfer of triboelectric charges in the asymmetric electrodes, which is used to continuously change the shape of the air–liquid interface between concave and convex without any complicated boost converter. The performance of the proposed TVLL was investigated in detail for different structural parameters; when the electrode width ratio was 0.1 and the volume of the optical medium in the liquid lens was 20 μL, the focal length was modulated from −6 mm to infinite to 7 mm by sliding the TENG. In addition, a triboelectric magnifying glass was demonstrated based on the accurate modulation effect of the TVLL on a light beam, in which the magnification ratio could be directly modulated by sliding the triboelectric layer of the TENG. In this work, the TENG was used as a medium to modulate and accurately control the focal length of a liquid lens by external mechanical sliding, which may have great application potential in MOEMS, human–machine interaction, artificial vision systems, etc.

## Materials and methods

### Etching of the micro/nanostructures on the surface of FEP

First, an FEP (10 μm thick) film was cleaned by alcohol and deionized water within an ultrasonic cleaner. Next, a thin film layer of copper was deposited on the cleaned FEP film by magnetron sputtering (Denton Vacuum Discovery-635). Then, ICP etching (SENTECH SI-500) was performed, where the flow rates of O_2_, Ar, and CF_4_ gases were 20.0, 30.0, and 35.0 standard cubic centimeters per minute, respectively, and the power of the plasma source was 500 W. The etching process lasted 60 s.

### Fabrication of the TVLL

To fabricate the freestanding-mode TENG, two pieces of acrylic (250 × 190 mm and 250 × 90 mm) were first cut as substrates by a laser cutter (Universal PLS6.75) with a cutting depth of 5 mm. Then, a 10 μm-thick copper film was adhered on one of the acrylic pieces (250 × 190 mm) and cut into two pieces. Next, the prepared micro/nanostructured FEP film was adhered on the other acrylic piece (250 × 90 mm) to form the sliding friction layer. The liquid lens was fabricated by common lift-off fabrication technology; the detailed fabrication procedure is shown in Fig. S1 and the supporting information.

### Characterization of the TVLL

The morphology of the FEP surface was observed by scanning electron microscopy (Nova NanoSEM 450). The sliding distance of the TENG was controlled by a linear motor (Akribis System). The open-circuit voltage was measured by an electrostatic meter (Trek 347), and the transferred charges of the TENG were measured by a preamplifier electrometer (Keithley 6514). The capacitance was measured by an LCR meter (Agilent 4263B). The focal length of the liquid lens was measured by the constructed testing system shown in Fig. S2. Photos and video were taken by a digital camera (Canon EOS 5D Mark III).

## Tribotronics Research Group

### Brief introduction

The Tribotronics Research Group mainly focuses on nanogenerators (NGs) and self-powered microsystems, in particular triboelectric nanogenerators (TENGs), tribotronics, and self-powered MEMS/NEMS. In recent years, a series of original and influential works have been reported, including on the mechanism characterization, micro/nano-fabrication, and application of TENGs.

The group revealed the symmetry and complementarity between a TENG and a traditional electromagnetic generator (EMG) for the first time (*Adv. Mater.* 26, 3580–3591 (2014)) and developed enhanced TENGs with ultrahigh surface charge density and output power (*Nano Energy* 49, 625–633 (2018); *Nano Res.* 8, 722–730 (2015)).

The group initiated the new field of tribotronics by coupling triboelectricity and semiconductors and carried out a series of influential works in fundamental theory, material diversity, functional devices, and system integration of tribotronics (*Nano Today* 11, 521–536 (2016); *ACS Nano* 8, 8702–8709 (2014)). Meanwhile, they also proposed a universal power management module for TENGs, which broke through the bottleneck of low supply efficiency and provided a complete TENG-based microenergy solution for self-powered intelligent systems (*Nano Energy* 37, 168–176 (2017)).

The group also made significant progress in the application of TENGs in the fields of robot touch (*Adv. Mater.* 30, 1800066 (2018)), wearable devices (*Nano Energy* 31, 533–540 (2017)), self-powered MEMS/NEMS (*Nat. Commun.* 10, 2309 (2019)*; Adv. Mater.* 27, 719–726 (2015)), wireless sensing (*Nano Energy* 61, 1–9 (2019)), etc.

## Supplementary information

Supporting information

Video S1

Video S2
