# Supplementary material for: Triboelectric effect-modulated varifocal liquid lens
Source: Microsyst Nanoeng. 2020 Aug 10;6:61. doi: 10.1038/s41378-020-0174-y (PMC8433165; doi:10.1038/s41378-020-0174-y)
Supplement: Supplementary file 1 — Supporting information [file 41378_2020_174_MOESM1_ESM.docx]

**Support information**

**Triboelectric Effect-Modulated Varifocal Liquid Lens**

Chunlong Fang^1,2^, Yuanzhi Cao^1^, Dongdong Jiang^1^, Jiarui Tian^1^ and Chi Zhang^1,2,3,^ **^*^**

^1^CAS Center for Excellence in Nanoscience, Beijing Key Laboratory of Micro-nano Energy and Sensor, Beijing Institute of Nanoenergy and Nanosystems, Chinese Academy of Sciences, Beijing, 100083, P. R. China

^2^School of Nanoscience and Technology, University of Chinese Academy of Sciences, Beijing, 100049, P. R. China

^3^Center on Nanoenergy Research, School of Physical Science and Technology, Guangxi University, Nanning, 530004, P.R. China

*Corresponding author: Chi Zhang, E-mail: [czhang@binn.cas.cn](mailto:czhang@binn.cas.cn)


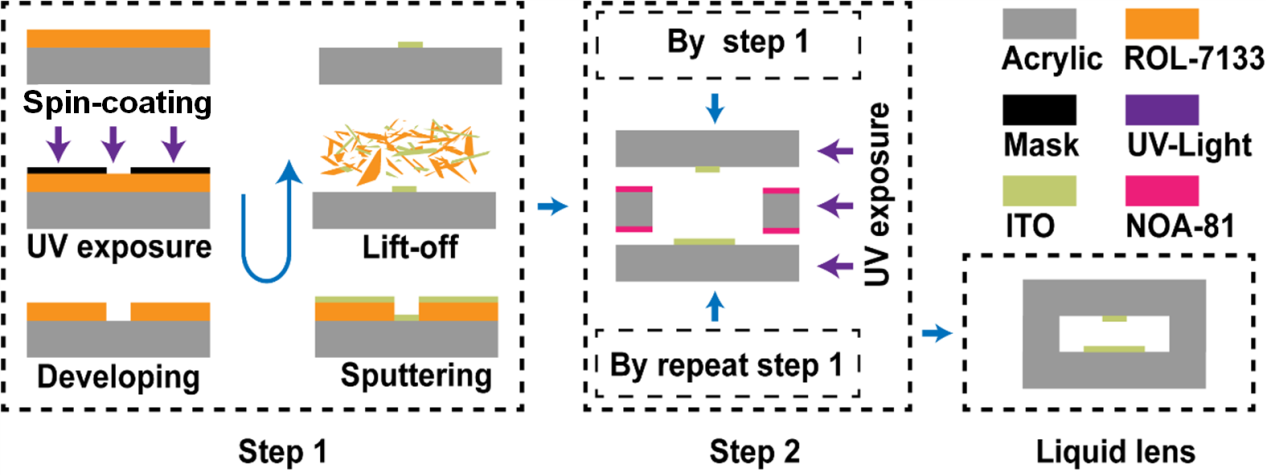


**Fig. S1. The fabrication procedure flow of the proposed TVLL**.

The fabrication procedure of the TVLL could be described as follows. At the first, the cut acrylic (with size of 3x3cm) substrate was cleaned with alcohol and deionized water in an ultrasonic cleaner. After that, the asymmetric electrode was made by the common lift-off technology (Fig. S1. Step 1). Then, the device assembled by the UV curing agent (NOA-81), as shown in Fig. S1. Step 2.


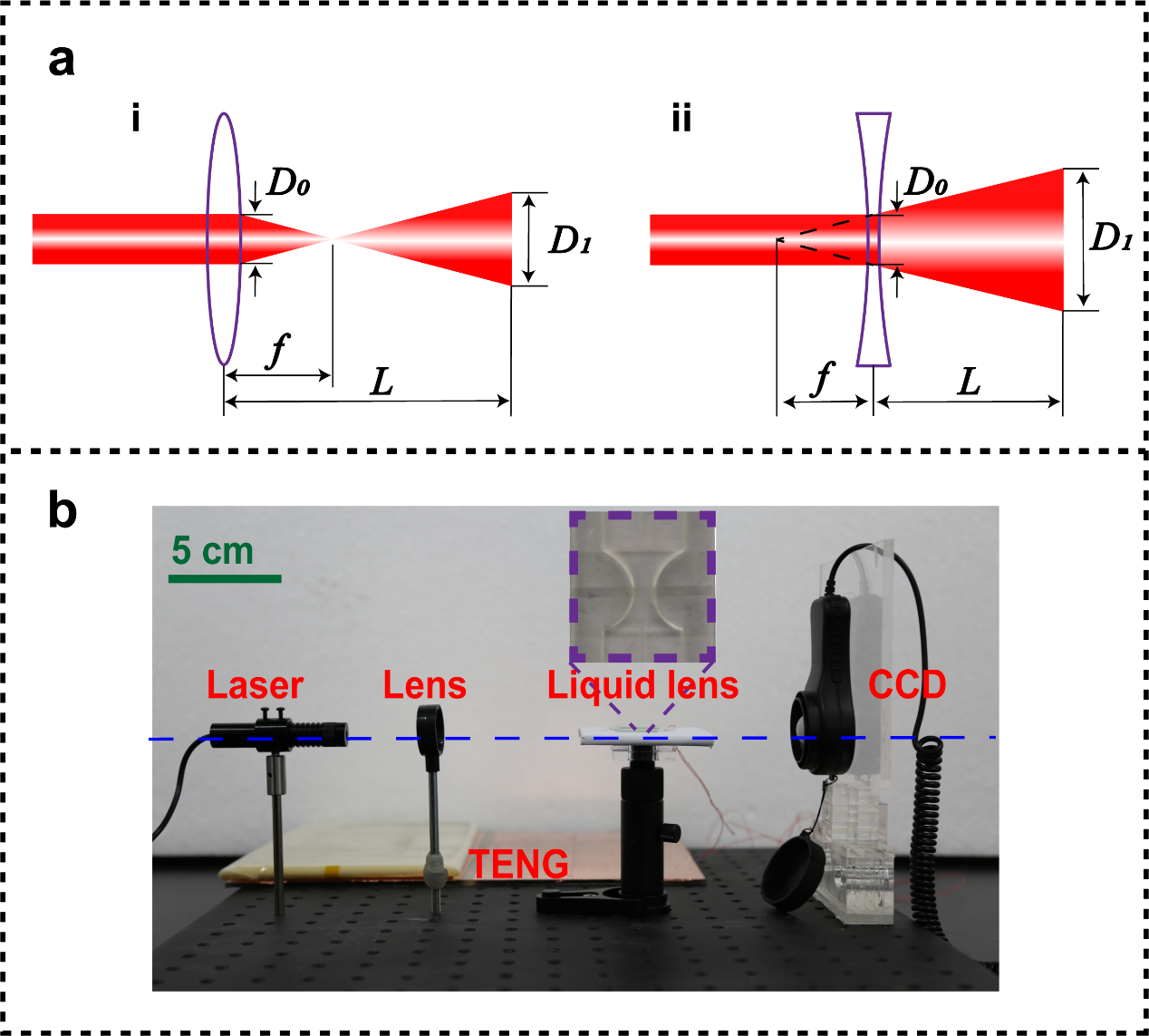


**Fig. S2. The constructed testing system for measuring focal length of the TVLL. a** The principle of the testing. **b** The photograph of the testing system.

The principle of the testing method of focal length as follows:

For a convex lens, as the schematic diagram shown in Figure S2. I. According to geometrical optics, the focal length (*f)* could be calculated by,

$f= \frac{D_{0}}{D_{0}+D_{1}}L$ (S1)

Where *D_0_* is the diameter of the incident light beam, *D_1_* is the diameter of the outgoing light beam at *L* from the center of the lens (*L*>*f*).

Similarly, for a concave lens (Fig. S2. II.). The focal length (*f)* could be calculated by,

$f= \frac{D_{0}}{D_{1}-D_{0}}L$ (S2)

The *D_0_* and *D_1_* measured by a CCD, and the *L* obtained by a precision distance measuring equipment.


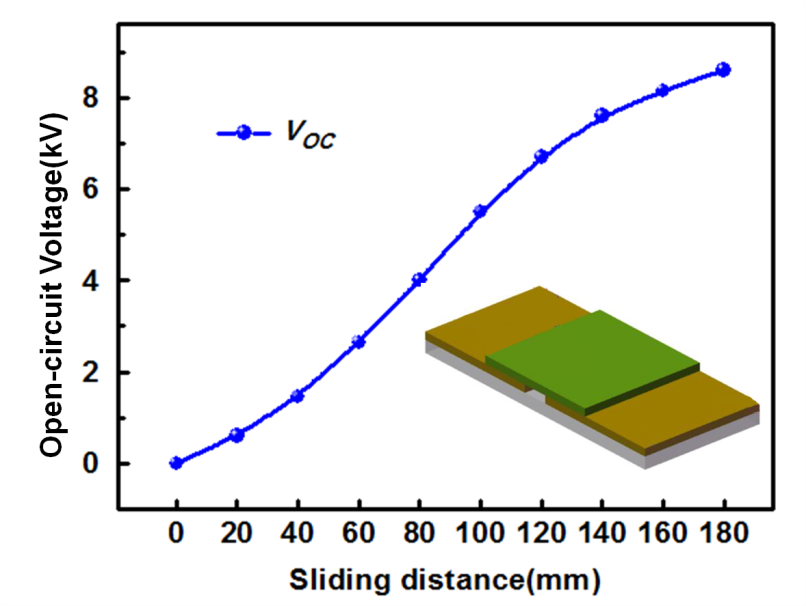


Fig. S3. The simulated open-circuit voltage(*V*_OC_) vs. sliding distance. The insert is the schematic diagram of the TENG.

The open-circuit voltage(*V*_OC_) vs. sliding distance is simulated by using the finite element method (FEM) in the COMSOL 5.4 software. As illustrated in Fig. S3, the open-circuit voltage increases monotonically with the increase of the sliding distance in the TENG, which is well agreement with the experimental measurement(Fig. 3a) and previous work.
